# Research Trends, Knowledge Evolution and Pharmacological Advances of *Cordyceps cicadae*: A Bibliometric Analysis

**DOI:** 10.3390/ijms27219574

**Published:** 2026-10-26

**Authors:** Siyu Du, Ruyue Luo, Anqi Liu, Tongling Yang, Ningbo Wang, Jialin Duan

**Affiliations:** 1 School of Life Science and Technology, Northwestern Polytechnical University, Xi’an 710129, China; 15735936398@163.com (S.D.); 16697035060@163.com (R.L.); 15984335512@163.com (A.L.); yangtongling2000@163.com (T.Y.); 13653469310@163.com (N.W.); 2 Key Laboratory of Shaanxi Province for Craniofacial Precision Medicine Research, College of Stomatology, Xi’an Jiaotong University, Xi’an 710049, China

**Keywords:** *C. cicadae*, scientometric, polysaccharides, antioxidant activity, immunomodulation, traditional Chinese medicine

## Abstract

*Cordyceps cicadae* (*C. cicadae*), an entomopathogenic medicinal fungus widely used in traditional Chinese medicine, has attracted growing scientific interest due to its diverse reported pharmacological activities. Despite increasing research activity, a comprehensive bibliometric assessment of global publication trends and research hotspots remains limited. This study aimed to analyze publication trends, research characteristics, and thematic developments in English-language publications on *C. cicadae* indexed in the Web of Science Core Collection (WoSCC). Relevant publications covering the period 1990–2025 were retrieved, yielding 192 eligible records, including 188 original articles and four reviews. Bibliometric analyses were performed using VOSviewer(version 1.6.20), CiteSpace(version 6.3.R1), and Bibliometrix/Biblioshiny(version 4.3.3) to evaluate publication output, contributing countries, authors, journals, co-citation networks, keyword co-occurrence patterns, and thematic evolution. Annual publication output remained relatively low in the early years but increased markedly after 2018. China accounted for the largest proportion of publications, while researchers such as Chen Chin-Chu, Li Wei, and Ouyang Zhen were among the notable contributors. Co-citation and keyword analyses identified the isolation and characterization of bioactive constituents and the investigation of pharmacological activities as major research themes. More recent research topics included immunomodulation, antioxidant activity, renal and hepatoprotective effects, gut microbiota modulation, and multi-omics-based mechanistic investigations. Overall, this study provides a systematic overview of the research landscape of *C. cicadae* and identifies emerging topics that may inform future investigations into cultivation strategies, bioactive constituents, pharmacological mechanisms, and potential biomedical applications.

## 1. Introduction

Among medicinal fungi traditionally classified within the genus *Cordyceps*, *Cordyceps sinensis* has long been recognized as a highly valued medicinal resource in traditional Chinese medicine due to its reported pharmacological properties and long history of medicinal use [1]. However, its natural distribution is largely confined to the high-altitude regions of the Qinghai–Tibet Plateau, and limited wild resources, growing market demand, and challenges associated with large-scale artificial cultivation have contributed to resource scarcity and high market prices [2]. These limitations have stimulated interest in exploring other medicinal fungi with potentially similar pharmacological properties.

*Cordyceps cicadae (Miq.)* Massee is an entomopathogenic fungus that infects cicada nymphs and develops characteristic flower-like stromata emerging from the infected hosts [3,4].

According to Species Fungorum and MycoBank, *Cordyceps cicadae* (Miq.) Massee and *Paecilomyces cicadae* (Miq.) Samson are listed as synonyms of *Isaria cicadae* Miq. Li et al. [5] conducted morphological examinations and multilocus phylogenetic analyses, revealing that *Isaria (Paecilomyces) cicadae* represents a species complex and identifying *Cordyceps chanhua* as a distinct species within the family Cordycipitaceae. This medicinal fungus is traditionally known as “Chan Hua” (cicada flower) in China [6]. Previous studies have reported that *C. cicadae* and *C. sinensis* share several bioactive constituents and exhibit overlapping pharmacological activities, suggesting that *C. cicadae* may have potential as an alternative medicinal resource, although their therapeutic equivalence has not been established [7]. Table 1 summarizes the similarities and differences between *C. cicadae* and *C. sinensis* in terms of their active constituents, pharmacological activities, and potential clinical applications.

Historically, *C. cicadae* has been used in traditional Chinese medicine for over a millennium. Classical Chinese medical texts, including Lei Gong’s Treatise on Preparation and the Compendium of Materia Medica, describe its traditional uses for improving vision, clearing wind-heat, alleviating convulsions, and managing childhood ailments [11,12]. Modern phytochemical investigations have identified a wide range of potentially bioactive constituents in *C. cicadae*, including polysaccharides [13], nucleosides [14], sterols [15], cyclic peptides, and other secondary metabolites. These constituents have been reported to exhibit a broad spectrum of pharmacological activities, including antioxidant, immunomodulatory, anti-inflammatory, anti-aging, antitumor, hypoglycemic, hepatoprotective, and renoprotective effects, suggesting their potential relevance to future pharmacological research and therapeutic development [16,17,18,19,20].

Advances in natural product chemistry, pharmacology, biotechnology, and fungal cultivation have contributed to the growth of research on *C. cicadae* over the past decade. The observed increase in publications suggests growing scientific interest in its bioactive constituents, pharmacological mechanisms, cultivation technologies, and potential therapeutic applications. However, the existing literature remains relatively fragmented, with individual studies primarily addressing specific aspects of this medicinal fungus. To our knowledge, a comprehensive bibliometric analysis examining the knowledge structure, collaboration networks, research evolution, and emerging themes of *C. cicadae* research has not yet been reported.

Bibliometric analysis provides a quantitative approach to evaluating scientific publications and characterizing the intellectual structure and development of research fields [21]. By combining statistical analysis with scientific knowledge visualization, this approach facilitates the identification of influential authors, institutions, journals, collaborative networks, research hotspots, and emerging trends. VOSviewer is commonly used to visualize bibliometric networks, including keyword co-occurrence and co-citation relationships, while CiteSpace supports the analysis of knowledge evolution and the detection of emerging research topics [22,23]. In addition, Bibliometrix and its web-based interface, Biblioshiny, provide integrated tools for bibliometric performance analysis and thematic exploration.

Accordingly, the present study conducted a comprehensive bibliometric analysis of research publications on *C. cicadae* indexed in the Web of Science Core Collection (WoSCC) covering the period 1990–2025. Using VOSviewer, CiteSpace, and Bibliometrix/Biblioshiny, we examined publication trends, research contributions, collaboration networks, co-citation relationships, keyword co-occurrence patterns, and thematic evolution. By systematically characterizing the research landscape and identifying major thematic developments, this study aims to provide an evidence-based overview of the field and inform future investigations into cultivation strategies, bioactive constituents, pharmacological mechanisms, and potential biomedical applications of *C. cicadae*.

## 2. Results

### 2.1. Trends in Publication Output

A total of 192 publications related to *Cordyceps cicadae* were retrieved from the Web of Science Core Collection, involving 931 authors from 265 institutions across 20 countries.

**Figure 1:**
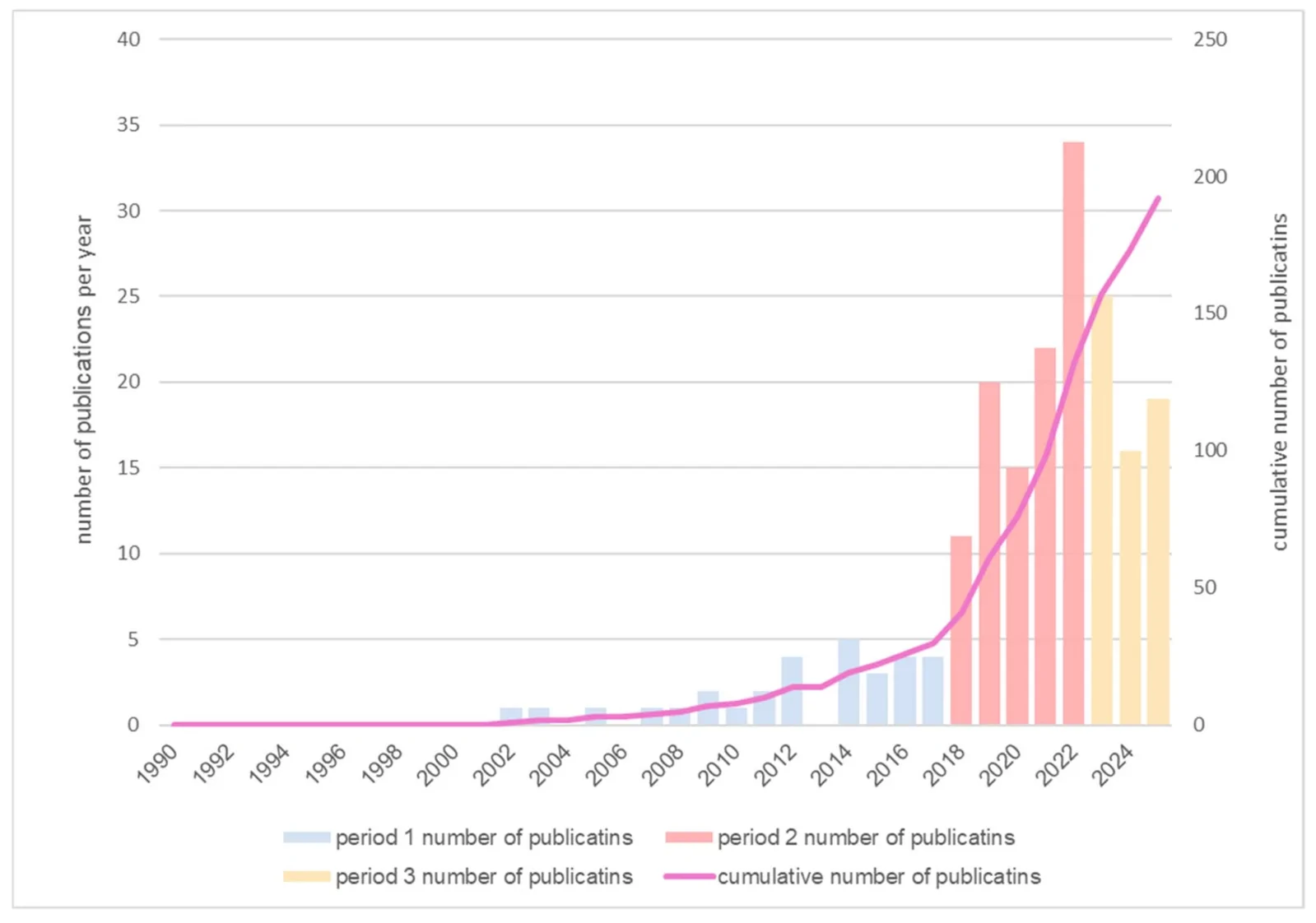
The bar graph shows the number of publications related to *Cordyceps cicadae* per year from 1990 to 2025. The line graph shows the cumulative number of publications each year.

Figure 1 presents the annual publication trends. From 1990 to 2017, the number of publications was relatively low, with fewer than five articles published per year in most years, indicating that C. cicadae research was still in its early stages. However, since 2018, the annual publication volume has increased significantly, rising from 11 articles in 2018 to a peak of 34 articles in 2022. Although the annual output decreased slightly after 2022, it continued to be more than 15 articles per year, reflecting the continued research interest in this field. Consistent with these annual publication trends, the cumulative publication curve shows an overall upward trend in research output throughout the study period.

### 2.2. Country/Region Analysis

**Figure 2:**
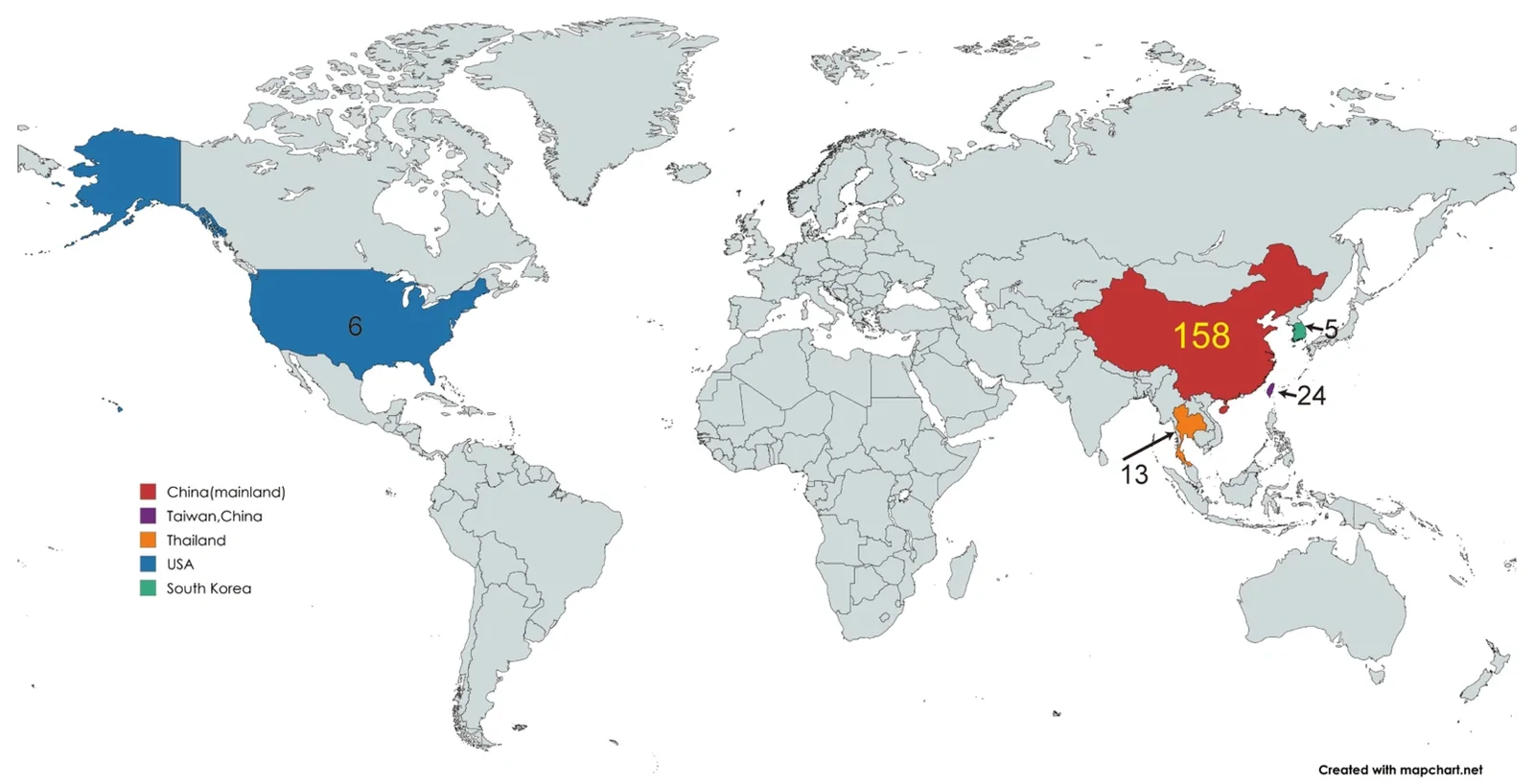
Global distribution map of the major contributing countries/regions. Figure generated using MapChart (https://www.mapchart.net). The numbers indicate the publication counts of the major contributing countries/regions.

As shown in Figure 2, the geographical distribution of research output in this field is mainly concentrated in Asia (China, Thailand, South Korea) and the United States. From the perspective of the number of published papers, China has the most papers, with a total of 182 papers, including 158 from mainland China and 24 from Taiwan (China), accounting for the majority of the total output. Thailand ranks second with 13 papers; the United States ranks third with 6 papers. These results indicate that China was the leading contributor to *C. cicadae* research in terms of publication output.

### 2.3. Author Collaboration Analysis

Author productivity analysis was performed to identify the core contributors to *Cordyceps cicadae* research. Table 2 presents the distribution of author productivity within the dataset. The results reveal a highly skewed distribution pattern. The vast majority of authors (76.6%, *n* = 713) contributed only a single document. The number of authors declines sharply as the number of documents written increases. Only 14.5% (*n* = 135) of authors published two documents, and the proportion drops below 5% for those publishing three or more documents. This distribution indicates that author productivity is highly uneven. The most productive authors are summarized in Table 3. Chen Chin-Chu ranked first with 15 publications, which received a total of 394 citations (26.3 citations per publication on average). His research mainly focused on the pharmacological effects of *C. cicadae* in diabetes, ocular diseases, and renal disorders [24,25,26]. Ouyang Zhen was the author with the highest average citation, and his research mainly focused on the antioxidant [20], neuroprotective [27] and anti-aging [28] effects of the related components of *C. cicadae*.

**Figure 3:**
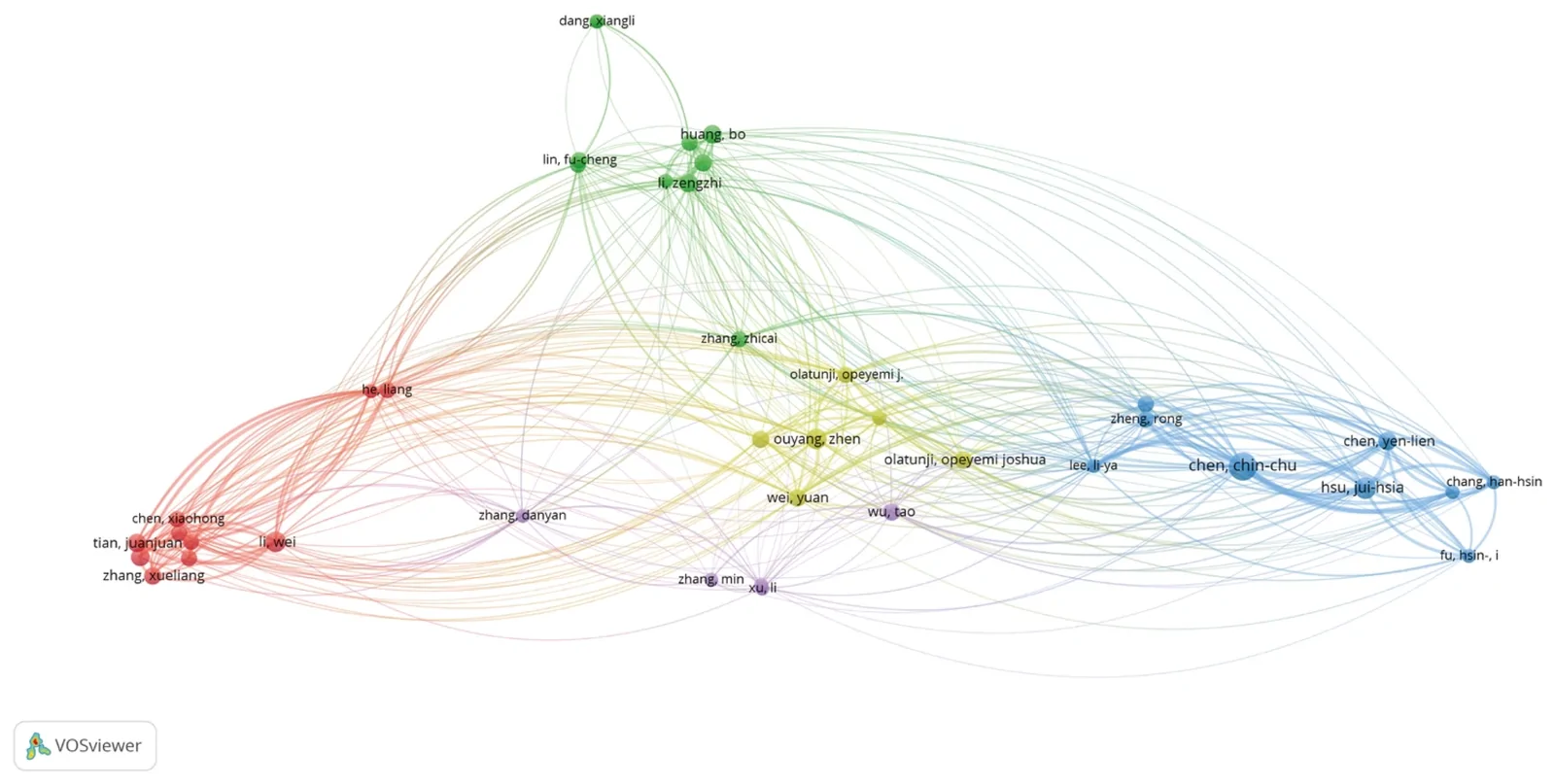
Knowledge domain map of co-authors of *Cordyceps cicadae* research studies. Node size is proportional to the number of co-authored publications; Different colors (red, green, blue, yellow, and purple) indicate distinct co-authorship clusters identified by VOSviewer; thicknesses of connecting lines indicate frequencies of collaboration.

The co-authorship network generated by VOSviewer (Figure 3) comprised 5 collaboration clusters, representing multiple research teams with varying degrees of cooperation. Most collaborations occurred among researchers within the same institutions, whereas collaborations between different institutions were comparatively limited. International collaboration was observed but remained relatively sparse, suggesting that cooperation across countries has yet to be further expanded.

### 2.4. Journal Analysis

A total of 192 publications on *Cordyceps cicadae* were distributed across 110 academic journals, indicating that research in this field has been disseminated through a wide range of disciplines. Most publications appeared in journals related to pharmacology, medicinal fungi, food science, natural products, and biotechnology, reflecting the interdisciplinary characteristics of *C. cicadae* research.

**Figure 4:**
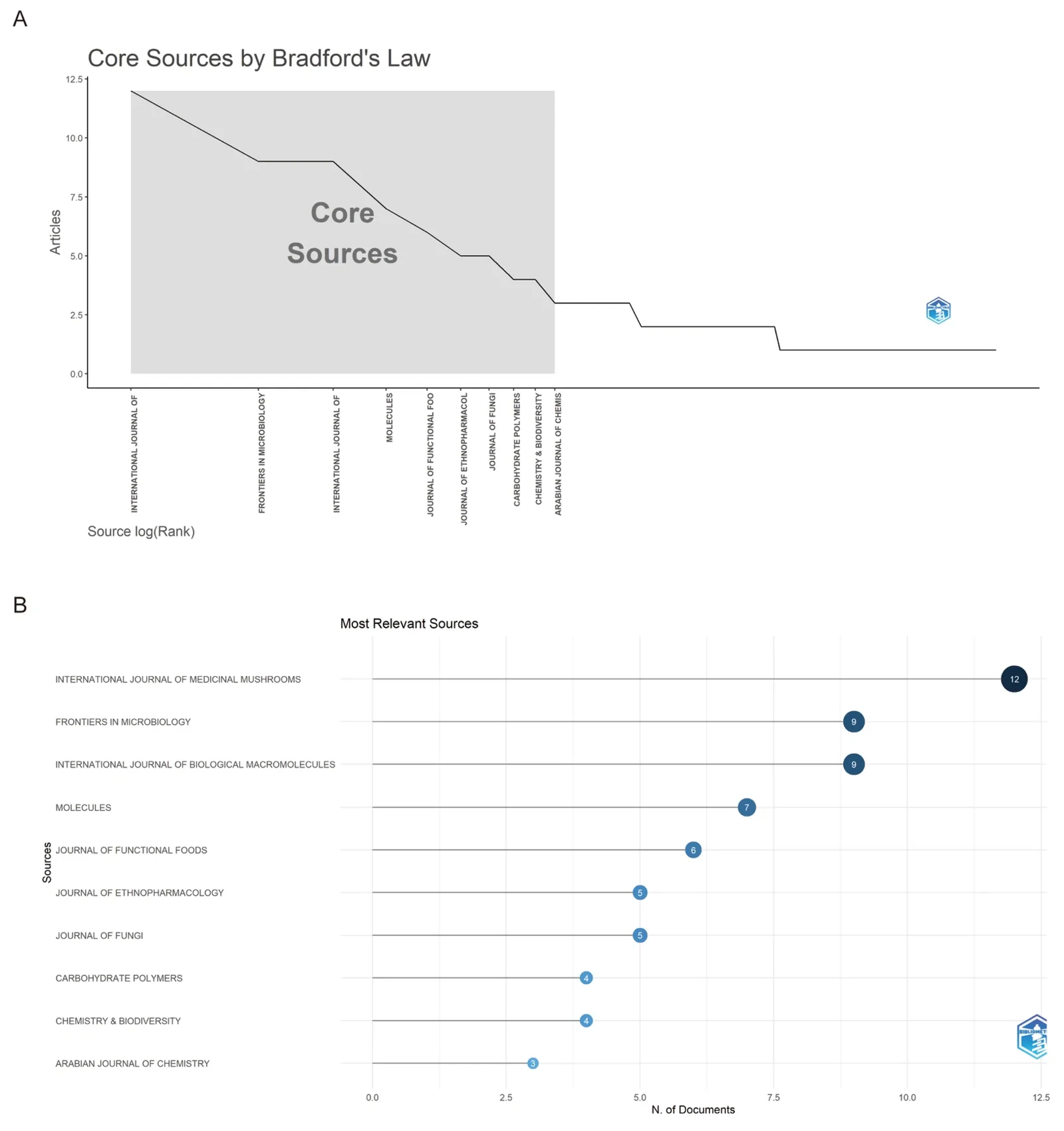
Analysis of journals. (**A**) 10 core journals based on Bradford’s Law. (**B**) The top 10 journals in terms of the number of published papers. Each blue circle represents a journal, the number inside indicates its publication count, and darker blue indicates a higher publication count.

According to Bradford’s Law, 10 journals were identified as core journals (Figure 4A). However, due to the limited dataset size (192 papers across 110 journals), the zones are relatively thin, with an average of only 1.7 papers per journal across the entire dataset. The zone distribution is presented in Table 4.

The distribution of the most productive journals is presented in Table 5 and Figure 4. The International Journal of Medicinal Mushrooms ranked first with 12 publications and a total of 142 citations, making it the leading publication venue for *C. cicadae* research. Although the International Journal of Biological Macromolecules published only nine articles, it achieved the highest average citation rate (48.2 citations per publication), indicating the relatively high academic impact of studies published in this journal.

Analysis of publications in the core journals revealed three major research themes: (i) extraction, purification, and structural characterization of polysaccharides and other bioactive constituents; (ii) pharmacological activities and molecular mechanisms underlying the therapeutic effects of *C. cicadae*; and (iii) optimization of cultivation, fermentation, and strain improvement technologies. These topics represent the principal research directions published in the leading journals of this field.

### 2.5. Co-Citation Analysis

**Figure 5:**
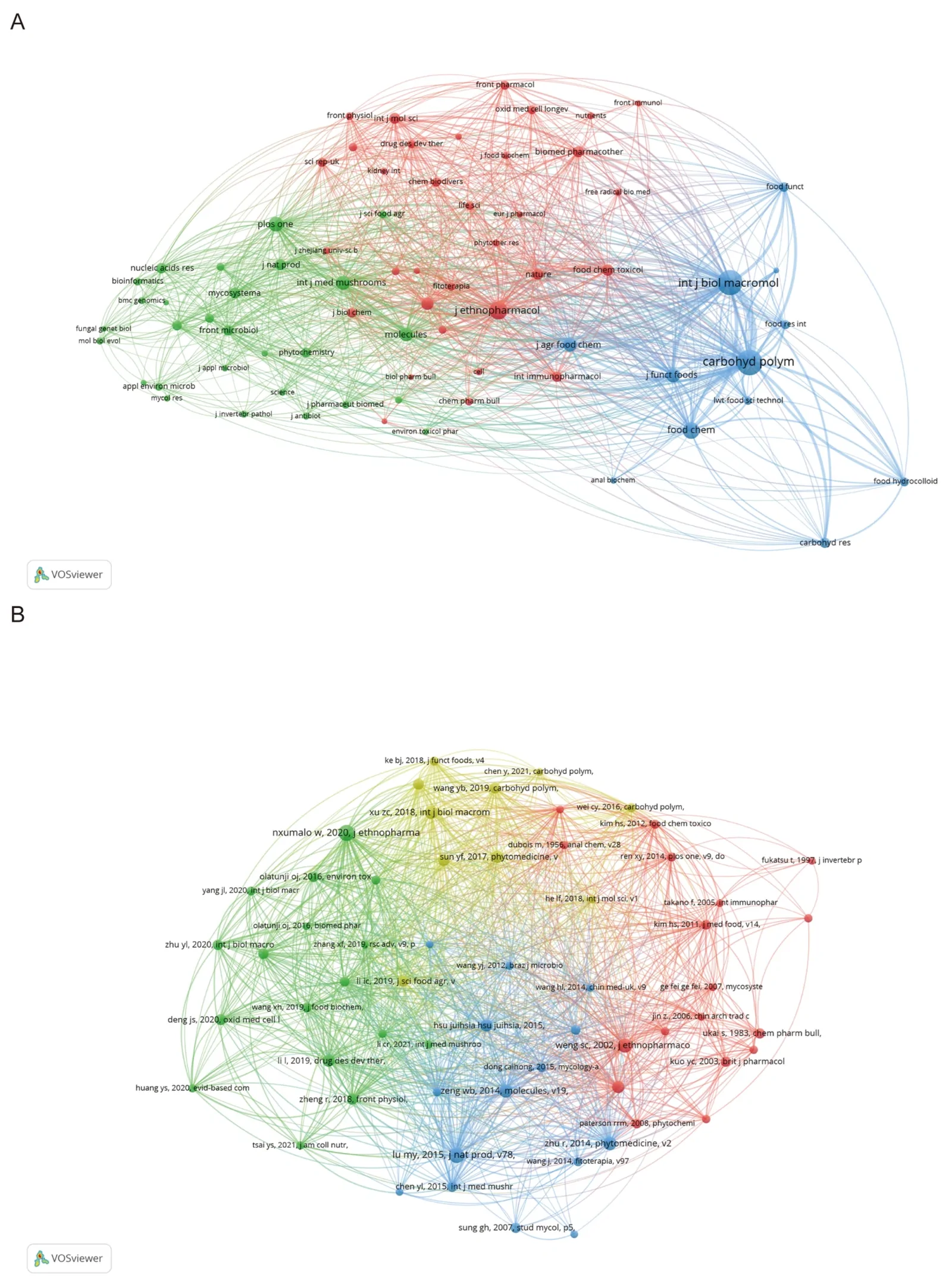
Co-citation analysis. (**A**) The co-citation network among journals. (**B**) The co-citation network among references.

Co-citation analysis was performed to identify the knowledge base and intellectual structure of *Cordyceps cicadae* research. Journal co-citation analysis revealed three major disciplinary clusters (Figure 5A), corresponding primarily to pharmacology (red), fungi and microbiology (green), and biological macromolecules (blue). The size of each node represents the co-citation frequency of the corresponding journal. Among all journals, Carbohydrate Polymers (332 co-citations), International Journal of Biological Macromolecules (316 co-citations), and Journal of Ethnopharmacology (170 co-citations) were the most frequently co-cited, indicating that these journals constitute the primary knowledge sources supporting *C. cicadae* research.

To further characterize the intellectual structure of the field, reference co-citation analysis was performed using VOSviewer. A threshold of at least 10 citations per reference was applied, resulting in 60 highly co-cited references being included in the final network (Figure 5B). The five most frequently co-cited publications are listed in Table 6. The red cluster was mainly involved in immunoregulation and early activity studies. The blue cluster focuses on nucleoside compounds, particularly N6-(2-hydroxyethyl) adenosine (HEA). Research in this cluster mainly explored the biological activities and molecular mechanisms of HEA, highlighting its anti-inflammatory and organ-protective properties. The green cluster mainly includes studies investigating the pharmacological mechanisms of *C. cicadae* extracts and other active compounds. These studies emphasized renal protection, antioxidant activity, and signal transduction pathways involved in disease intervention. The yellow cluster is primarily associated with polysaccharide research, including extraction, purification, structural characterization, and biological activities. Structure–activity relationships constitute the principal research topics within this cluster.

Overall, the reference co-citation network demonstrates that *C. cicadae* research encompasses the extraction and characterization of bioactive components, as well as in-depth exploration of pharmacological activities and disease intervention mechanisms. These highly co-cited publications constitute the principal knowledge base of the field and provide the theoretical foundation for subsequent research.

### 2.6. Keyword Analysis and Research Trends

**Figure 6:**
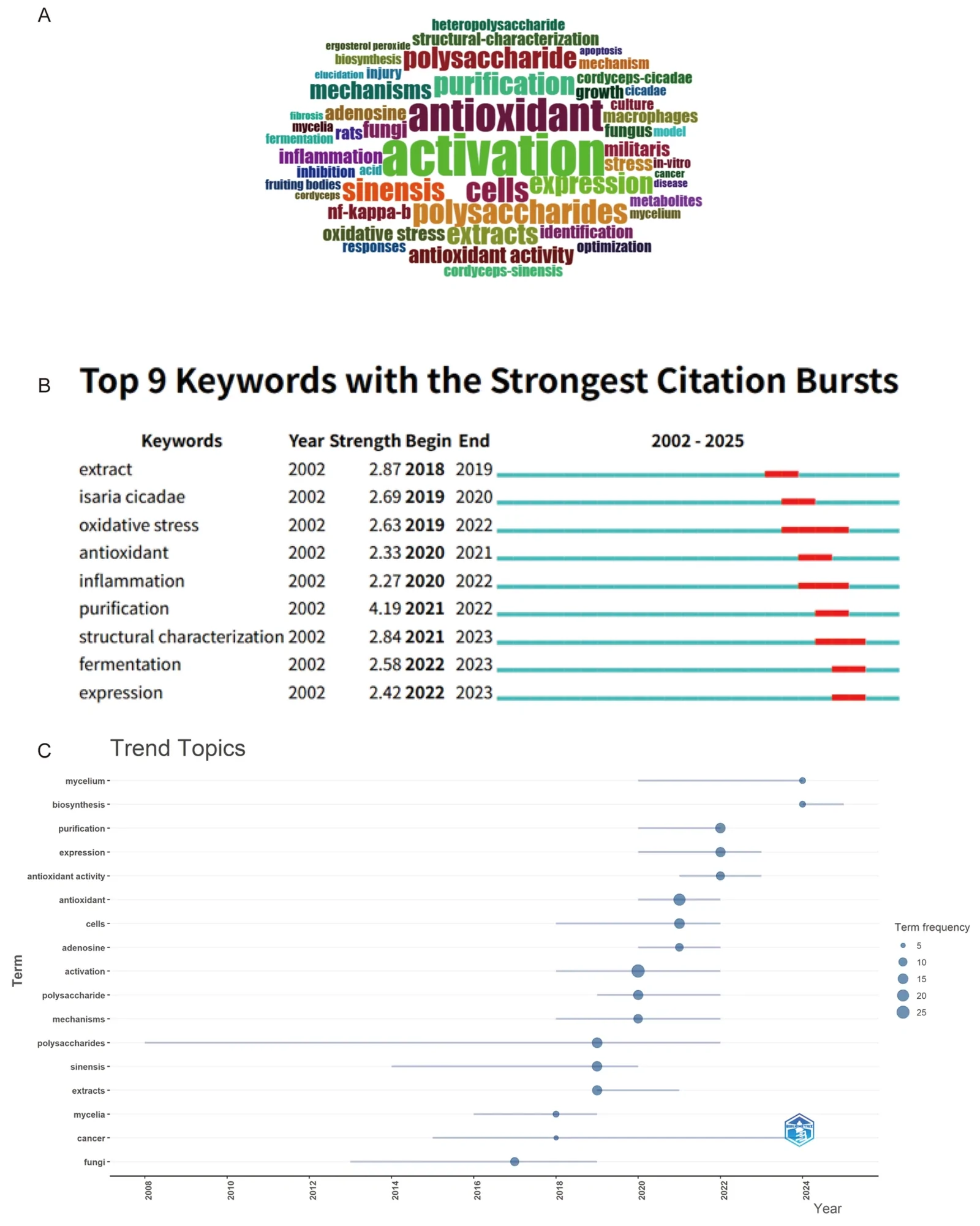
Keyword analysis. (**A**) WordCloud. (**B**) Top 9 keywords with the strongest citation bursts. Red segments indicate the burst periods of the corresponding keywords. (**C**) The trend topics.

High-frequency keywords were analyzed to identify the major research topics in *Cordyceps cicadae* research. The keyword word cloud is presented in Figure 6A, in which larger font sizes indicate higher keyword frequencies. Among these, polysaccharides, antioxidant, and immunomodulation were the most frequently occurring terms, indicating that bioactive constituents and their pharmacological activities constitute the principal research focus of this field.

Keyword burst analysis was performed to identify emerging research interests during the study period (Figure 6B). Nine burst keywords were detected, including extract, *Isaria cicadae*, oxidative stress, antioxidant, inflammation, purification, structural characterization, fermentation, and expression. Purification (burst strength = 4.19), extract (2.87), and structural characterization (2.84) exhibited relatively strong citation bursts, suggesting increasing research attention to active constituent purification and structural characterization.

Trend topic analysis (Figure 6C) further revealed the dynamic evolution of research interests over time. Recent studies have increasingly focused on mycelium, purification, antioxidant activity, and adenosine, indicating growing attention to bioactive compound development, cultivation optimization, and mechanistic investigations.

Overall, keyword co-occurrence, burst detection and trend topic analysis consistently demonstrate the evolution of *C. cicadae* research from early studies on extraction of bioactive constituents toward mechanistic investigations of pharmacological activities and disease intervention. Purification and structural characterization of polysaccharides, antioxidant activity, immunomodulation, gut microbiota, and cultivation technologies have remained major research themes throughout the study period.

## 3. Discussion

The present study provides the first comprehensive bibliometric evaluation of global *Cordyceps cicadae* research from 1990 to 2025. By integrating publication characteristics, collaboration networks, co-citation analysis, keyword co-occurrence, and thematic evolution, this study systematically characterized the knowledge structure, developmental trajectory, major hotspots, and emerging frontiers of this field. The findings revealed a clear shift in *C. cicadae* research from early exploratory studies focusing on chemical constituent isolation and cultivation techniques toward mechanistic investigations and disease-oriented pharmacological research. This transition highlights the growing scientific interest in *C. cicadae* as a medicinal fungus and its potential applications in biomedical research.

One of the most notable findings of this study is the substantial increase in publications after 2018, reflecting growing scientific interest in *C. cicadae*. This trend may be associated with several factors. First, advances in artificial cultivation and fermentation technologies have improved the availability and quality of *C. cicadae*, potentially alleviating the limitations associated with scarce natural resources. Second, increasing global interest in medicinal fungi and natural products may have encouraged further investigations into their bioactive constituents and potential therapeutic applications. Third, developments in analytical technologies, including high-resolution chromatography, nuclear magnetic resonance spectroscopy, metabolomics, transcriptomics, and multi-omics integration, have facilitated a more comprehensive characterization of the chemical composition and underlying molecular mechanisms of *C. cicadae*. Collectively, these developments may have contributed to the observed growth in research activity. Although this trend suggests sustained scientific interest in *C. cicadae*, future publication patterns remain uncertain and will likely depend on continued technological advances, emerging research priorities, and the availability of research resources.

Country/region analysis revealed that China accounted for the largest proportion of publications on *C. cicadae* research during the study period (1990–2025). This leading role may be attributed, at least in part, to the long history of medicinal use of *C. cicadae* in traditional Chinese medicine, the availability of abundant germplasm resources, and sustained government support for the modernization of traditional Chinese medicine. Nevertheless, the co-authorship network analysis indicated that international collaboration remains relatively limited, with most research collaborations concentrated among institutions within China. Strengthening interdisciplinary and multinational collaborations may facilitate knowledge exchange, encourage methodological innovation, and increase the global visibility of *C. cicadae* research.

Co-citation and keyword analyses provided insights into the intellectual foundations and thematic evolution of research on *C. cicadae*. Early investigations primarily focused on the isolation and characterization of bioactive constituents and the assessment of their antioxidant activities. As research progressed, greater emphasis was placed on investigating the underlying pharmacological mechanisms using molecular biological approaches, particularly those involving the regulation of signaling pathways, immunomodulation, oxidative stress, and disease-related processes. More recently, advances in cultivation technologies, the optimization of fermentation processes, bioactive nucleosides such as N6-(2-hydroxyethyl)adenosine (HEA), and the modulation of gut microbiota have emerged as prominent research topics. Collectively, these findings suggest a gradual shift in *C. cicadae* research from descriptive studies of chemical constituents toward mechanism-focused investigations and research exploring potential biomedical applications.

The following pharmacological discussion provides a narrative interpretation of the clusters. Among the various bioactive constituents identified in *C. cicadae*, polysaccharides remain the most extensively investigated and represent the core research hotspot throughout the study period. Structural analysis summarized in Table 7 indicates that *C. cicadae* polysaccharides exhibit considerable diversity in molecular weight, monosaccharide composition, glycosidic linkage patterns, and branching structures.

**Table 7:** Structure and analytical technique of polysaccharides from *Cordyceps cicadae*.

Name	Molecular Weight/ Molecular Mass	Carbohydrate Composition	Structural Characteristic	Methods of Analysis	Ref.
PC-EPS	3.72 × 10^6^ Da	Man:Glu:Gal = 28.84:1:19.42	→6)-α-d-Manp (1→, →2)-β-d-Manp (1→, →4) -α-d-Manp (1→, →2) -α-d-Galf (1→, and →4) -α-d-Galp (1→	FT-IR, NMR, Methylation analysis	[29]
BSP	1.28 × 10^5^ Da	Ara:Gal:Glu:Xyl = 7.60:1.80:76.10:14.50	It contains a variety of glycosidic linkage modes such as →4,6)-Galp-(1→, →2,6)-Manp -(1→, →5) -Araf -(1→), and is composed of eight glycosidic residues.	NMR, Methylation analysis, FT-IR, GC	[30]
SPP	4.3 × 10^4^ Da, 4 × 10^3^ Da	Ara:Gal:Glu:Xyl:Man = 9.10:15.40:41.20:17.50:16.80	It has linkage modes such as Araf-(1→, Xylp-(1→, →5)-Araf-(1→) and is composed of 12 glycoside residues.	NMR, Methylation analysis, FT-IR, GC	[30]
PPP	9.03 × 10^5^ Da, 2.1 × 10^4^ Da	Ara:Gal:Glu:Xyl:Man = 4.50:11.90:62.20:10.70:10.70	It contains links such as Araf-(1→, Xylp-(1→, →5) -Araf -(1→), etc., consisting of 15 glycoside residues.	NMR, Methylation analysis, FT-IR, GC	[30]
CPA-1	not reported	Glu:Man:Gal = 47.7:22.9:29.4	not reported	FT-IR, GC	[31]
CPB-2	not reported	Glu:Man:Gal = 79.7:11.1:9.1	not reported	FT-IR, GC	[31]
JCH-1	3.09 × 10^4^ Da	Glu:Man:Gal = 41.75:33.67:24.58	not reported	FT-IR, GC, HPGPC	[32]
JCH-2	5.553 × 10^5^ Da	Glu:Man:Gal = 72.59:13.99:13.42	not reported	FT-IR, GC, HPGPC	[32]
CCSP-1	1.79 × 10^6^ Da	Man:Glu:Gal = 12.29:80.46:7.25	not reported	HPGPC, GC-MS, FT-IR	[33]
CCSP-2	5.74 × 10^4^ Da	Glu:Man = 94.27:5.73	not reported	HPGPC, GC-MS, FT-IR	[33]
CCSP-3	7.93 × 10^3^ Da	Xyl:Man:Glu:Gal = 22.28:2.05:63.40:12.27	not reported	HPGPC, GC-MS, FT-IR	[33]
Galactoglucomannan	2.33 × 10^4^ Da	Gal:Glu:Man = 5:1:4	The core structure is a (1 → 2)-α-D-mannan backbone, which is further branched at the O-6 positions along the main chain. (1 → 4)-α-D-Glcp, (1 → 2)-β-D-Galf, (1 → 2,6)-β-D-Galf, and terminal β-Galf were located at the side chains.	HPGPC, Methylation analysis, NMR	[34]
Pc0-1	1.8 × 10^7^ Da	Glu:Gal:Man:Ara = 8:5:4:1	The core structure is a 1,2-linked α-D-Manp backbone, with additional branches attached at the O-3 and O-6 positions. The inner part of the side chains comprises 1,4-linked α-d-Glcp and 1,4-linked 6-O-Me-α-d-Glcp residues. 1,2-linked β-Galf and minor 1,4-linked Arap and 1,3 or 4-linked Arap residues were occasionally linked at the outside of the side chains.	HPLC, IR, methylation analysis, NMR	[35]
IPS1	2.40 × 10^6^ Da	Man:Glu:Gal = 1.35:6.93:1.0	→4)-α-D-Glcp (1 → and →3,4)-α-D-Manp (1 → residues	HPLC, GC, FT-IR, methylation analysis, NMR	[36]
IPS2	6.79 × 10^5^ Da	Man:Glu:Gal = 2.04:1.0:1.87	→4)-α-D-Glcp-(1→, →3,4)-α-D-Manp-(1 → and → 2,6)-α-D-Manp-(1 → residues	HPLC, GC, FT-IR, methylation analysis, NMR	[36]
JCH-a1	6.07 × 10^4^ Da	Gal:Glu:Man = 0.89:1:0.39	not reported	HPLC, FT-IR, SEM	[37]

Such structural heterogeneity may be attributed, at least in part, to differences in fungal strains, cultivation conditions, extraction methods, purification procedures, and analytical techniques. Accumulating evidence suggests that variations in these structural features may influence biological activities, highlighting the importance of establishing reliable structure–activity relationships. However, despite substantial progress, the detailed structural characterization of many polysaccharides remains incomplete, particularly regarding their higher-order conformations and three-dimensional structures. Future studies integrating advanced analytical techniques with computational modeling approaches may help provide further insights into the molecular basis of their pharmacological activities.

**Figure 7:**
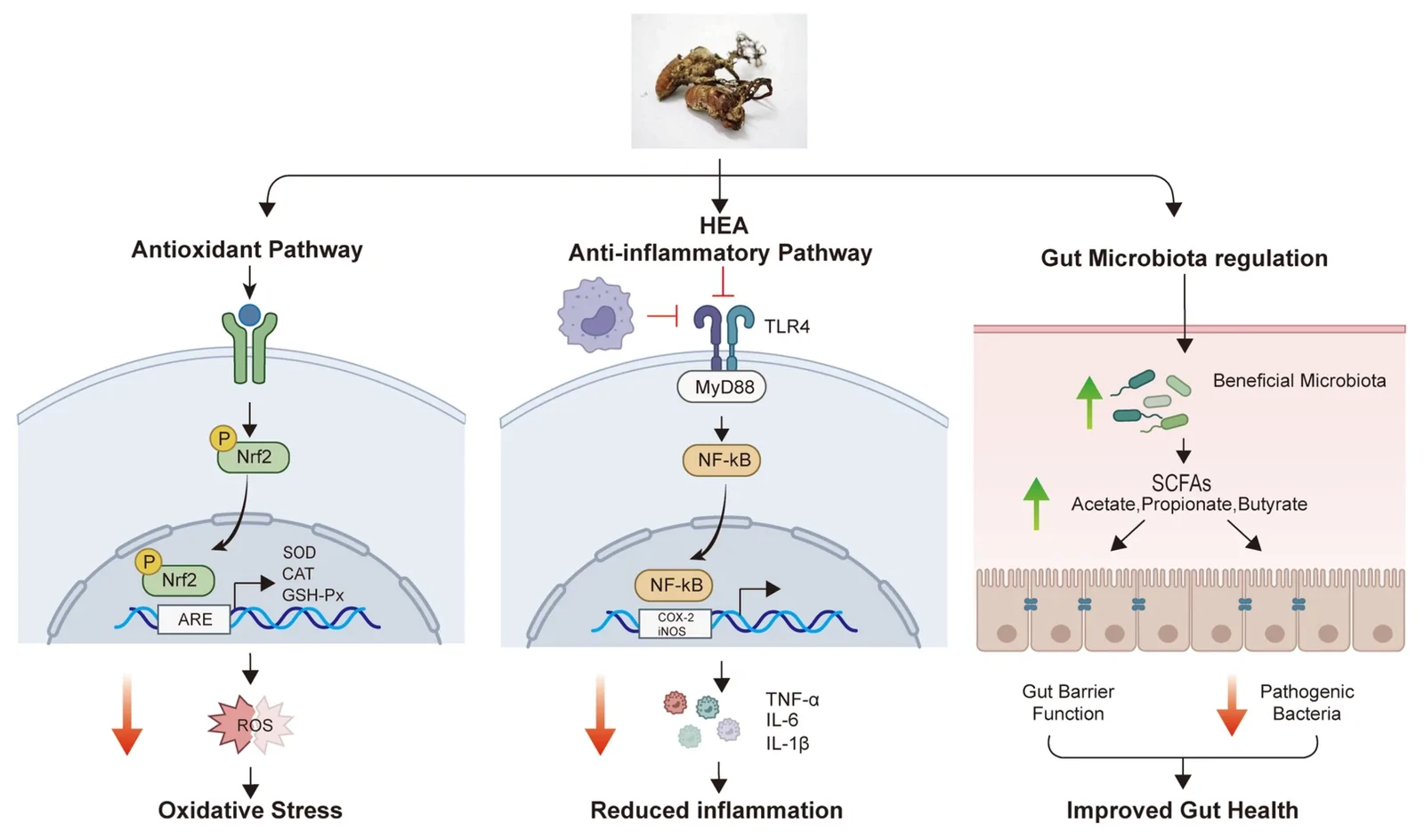
Mechanism by which *C. cicadae* improves host health through antioxidant, anti-inflammatory, and regulation of gut microbiota. Red downward arrows indicate reductions in oxidative stress, inflammation, or pathogenic bacteria, and green upward arrows indicate increases in beneficial gut microbiota and short-chain fatty acids (SCFAs).

Available pharmacological evidence suggests that immunomodulation, antioxidant activity, organ-protective effects, and modulation of the gut microbiota are among the major reported biological activities of *C. cicadae* (Table 8, Figure 7). Among these, immunomodulation appears to be an important mechanism contributing to its reported pharmacological effects. The immunomodulatory activities of *C. cicadae* vary depending on the bioactive constituents and experimental conditions. The spore polysaccharide CCSP-2 has been reported to enhance macrophage phagocytic activity, stimulate splenocyte proliferation, improve natural killer (NK) cytotoxicity, alleviate bone marrow suppression, regulate cytokine and immunoglobulin secretion, and modulate antioxidant enzyme activities, thereby alleviating cyclophosphamide-induced immunosuppression in mice [33]. Other polysaccharide preparations have been shown to activate macrophages through TLR4-mediated MAPK and NF-κB signaling [13]. In contrast, N6-(2-hydroxyethyl)adenosine (HEA) has been reported to inhibit TLR4-mediated NF-κB signaling, thereby reducing LPS-induced pro-inflammatory cytokine production [38]. Furthermore, a comparative study found no evidence of synergistic anti-inflammatory effects among adenosine, cordycepin, and HEA, with cordycepin exhibiting the highest potency among the three compounds. Additionally, both crude and indigestible polysaccharide fractions derived from cultivated *C. cicadae* fruiting bodies have been reported to exhibit anti-inflammatory activities [39].

In addition to immunomodulation, antioxidant activity represents another important pharmacological property of *C. cicadae*. Polysaccharides CPA-1 and CPB-2 have been reported to protect PC12 cells against glutamate-induced oxidative injury by improving cell viability and strengthening cellular antioxidant defenses [31]. Similarly, the polysaccharide component CP70 has demonstrated antioxidant and lifespan-extending effects in *Drosophila melanogaster*, accompanied by increased activities of catalase (CAT) and glutathione peroxidase (GSH-Px), and reduced malondialdehyde (MDA) levels [20]. Cordycepin has also been shown to attenuate neuronal cell death, apoptosis, and mitochondrial dysfunction, suggesting its potential neuroprotective properties [40]. Collectively, these findings suggest that the attenuation of oxidative stress may contribute to the reported anti-aging and neuroprotective effects of *C. cicadae* and its bioactive constituents.

Beyond its immunomodulatory and antioxidant activities, modulation of the gut microbiota may represent another important mechanism underlying the pharmacological effects of *C. cicadae*. In vitro simulated digestion experiments showed that *C. cicadae* polysaccharides were resistant to salivary and gastrointestinal digestion. Following in vitro fecal fermentation, however, their molecular weight decreased significantly, accompanied by marked changes in monosaccharide composition. These changes were associated with increased relative abundances of *Bacteroides* and *Phascolarctobacterium* and decreased relative abundances of *Escherichia-Shigella*, *Klebsiella*, and *Fusobacterium*. These observations suggest that *C. cicadae* polysaccharides can be utilized by intestinal microorganisms and may modulate gut microbial composition [41]. Similarly, administration of ultrasound-extracted *C. cicadae* polysaccharides to healthy mice for 42 days increased the relative abundances of *Lactobacillus*, *Akkermansia*, and *Bacteroides*, whereas *Clostridium* decreased. This treatment was also associated with increased expression of intestinal tight junction proteins [42]. Furthermore, in a mouse model of diabetes induced by a high-fat diet and streptozotocin (STZ), *C. cicadae* polysaccharides improved glycemic control, glucose tolerance, and insulin sensitivity, accompanied by alterations in gut microbial composition and metabolic profiles [43]. In addition to changes in microbial composition, emerging evidence suggests that gut microbiota-derived metabolites, particularly short-chain fatty acids and bile acids, may contribute to the interactions between intestinal microorganisms and host metabolic regulation [44]. Collectively, these findings suggest that gut microbiota–host interactions may provide a potential mechanistic basis for some of the reported pharmacological effects of *C. cicadae*. Future studies integrating microbiome profiling, metabolomics, and host signaling analyses may help clarify these complex interactions and their potential contributions to metabolic health.

**Table 8:** The representative pharmacological activities and mechanisms of *C. cicadae* constituents.

Bioactive Constituent	Pharmacological Activity	Model	Main Findings	Mechanism	Ref.
*C. cicadae*	Renoprotective	Spontaneously hypertensive rats with hypertensive nephropathy	Attenuate renal tubular injury and reduce TGF-β1, α-SMA, Col-1 and fibronectin expression	Regulation of autophagic stress through the SIRT1/FOXO3a pathway and oxidative stress modulation	[45]
Cordycepin	Antioxi-dant/neuroprotective	6-hydroxydopamine-induced injury in PC12 cells	Inhibit cell death, apoptosis, and mitochondrial dysfunction; decrease ROS and MDA levels and increase SOD and GSH-Px activities		[40]
*C. cicadae* cultured with deep-ocean water	Antioxidant/anti-aging	D-galactose-induced aging rats	Increase GRd and GPx; decrease aging-related protein		[46]
Exopolysaccharide	Immunomodulatory	Cyclophosphamide (CTX)-induced immunosuppressive mouse model; RAW 264.7 macrophages	Increase the immune organ indices, promote the secretion of serum immunoglobulins and upregulate the expression of cytokines; enhance the phagocytic activity and increase NO, iNOS, TNF-α, IL-6, IL-1β, IFN-γ and IL-4	Activation of TLR4/MyD88/NF-κB and MAPK signaling	[13,47]
cordycepin	Anti-inflammatory	LPS-stimulated RAW264.7 macrophages	Suppress pro-inflammatory cytokines		[38]
HEA	Anti-inflammatory	LPS-stimulated RAW264.7 macrophages	Suppress pro-inflammatory cytokines	Suppression of TLR4-NF-κB signaling	[38]
Exopolysaccharide	Gut microbiota modulation	Cyclophosphamide (CTX)-induced immunosuppressive mouse model	Increase the abundance of beneficial bacteria (*Muribaculaceae*, *Lachnospiraceae NK4A136*, *Bacteroides*, *Odoribacter*) and reduce the level of harmful bacteria (*Alistipes*, *Helicobacter*)	Modulation of gut microbial composition	[13]
polysaccharides from *C. cicadae* (CH-P)	Hypoglycemic; Gut microbiota modulation	High-fat diet/streptozocin-induced diabetic mice	Improve insulin sensitivity; reduce the *Firmicutes*/*Bacteroidetes* ratio and increase the abundances of *Bacteroides*, *Odoribacter*, *Alloprevotella*, *Parabacteroides*, *Mucispirillum*, and significantly decrease the abundances of *Helicobacter* and *Lactobacillus* compared to the diabetic group		[43]

Despite considerable advances in *C. cicadae* research over the past three decades, several important challenges remain. First, comprehensive and standardized quality control frameworks encompassing germplasm resources, cultivation conditions, processing procedures, and the quantification of bioactive constituents have yet to be fully established. Second, the available pharmacological evidence is predominantly based on in vitro studies and animal models, with relatively few high-quality clinical investigations. Bridging this gap will require more systematic evidence synthesis, standardized evaluation, and translational studies to determine whether promising preclinical findings can be reproduced and meaningfully evaluated in clinical settings [48]. Third, although a wide range of bioactive constituents have been identified, their possible interactions and the molecular mechanisms underlying their combined pharmacological effects remain insufficiently characterized. Finally, emerging technologies, including single-cell sequencing, spatial transcriptomics and integrated multi-omics approaches, may provide a more comprehensive view of the complex pharmacological mechanisms of *C. cicadae*. AI-assisted bioinformatics and computational modeling could further integrate these datasets, prioritize candidate compounds and pathways [49,50]. The application of these technologies could contribute to the identification of potential therapeutic targets and support further research into the development and biomedical applications of this traditional medicinal fungus.

This study has several limitations. Given the long history of *C. cicadae* use in traditional Chinese medicine, reliance on English-language publications indexed in the Web of Science Core Collection (WoSCC) may have resulted in the omission of relevant studies published in other databases, particularly Chinese-language literature. These database and language restrictions may have introduced selection bias and affected the representation of research trends and thematic patterns. In addition, publication and citation counts are influenced by the date of data retrieval, while indexing delays may lead to an underestimation of research output in the most recent years. The full counting method was applied to country/region and author analyses. Although this approach provides a straightforward measure of research participation, it assigns one full credit to each contributing country/region or author for a given publication, potentially overestimating the relative contributions of highly collaborative countries or authors. Finally, the discussion of pharmacological activities was based on a narrative synthesis of selected studies rather than a systematic review, and therefore may not capture the full scope of available pharmacological evidence.

## 4. Materials and Methods

### 4.1. Data Sources and Search Strategy

The bibliographic data analyzed in this study were retrieved from the Web of Science Core Collection (WoSCC). The search was conducted in WoSCC with “Editions: All” selected, covering all index editions available through our institutional subscription. The Web of Science Core Collection (WoSCC) is widely used in bibliometric research and provides bibliographic metadata and cited-reference information for publication and citation analyses [51,52]. The study focused on English-language articles and reviews indexed in WoSCC. CNKI and other databases were not included in this single-database design, which limits coverage, particularly of Chinese-language research on *C. cicadae*.

**Figure 8:**
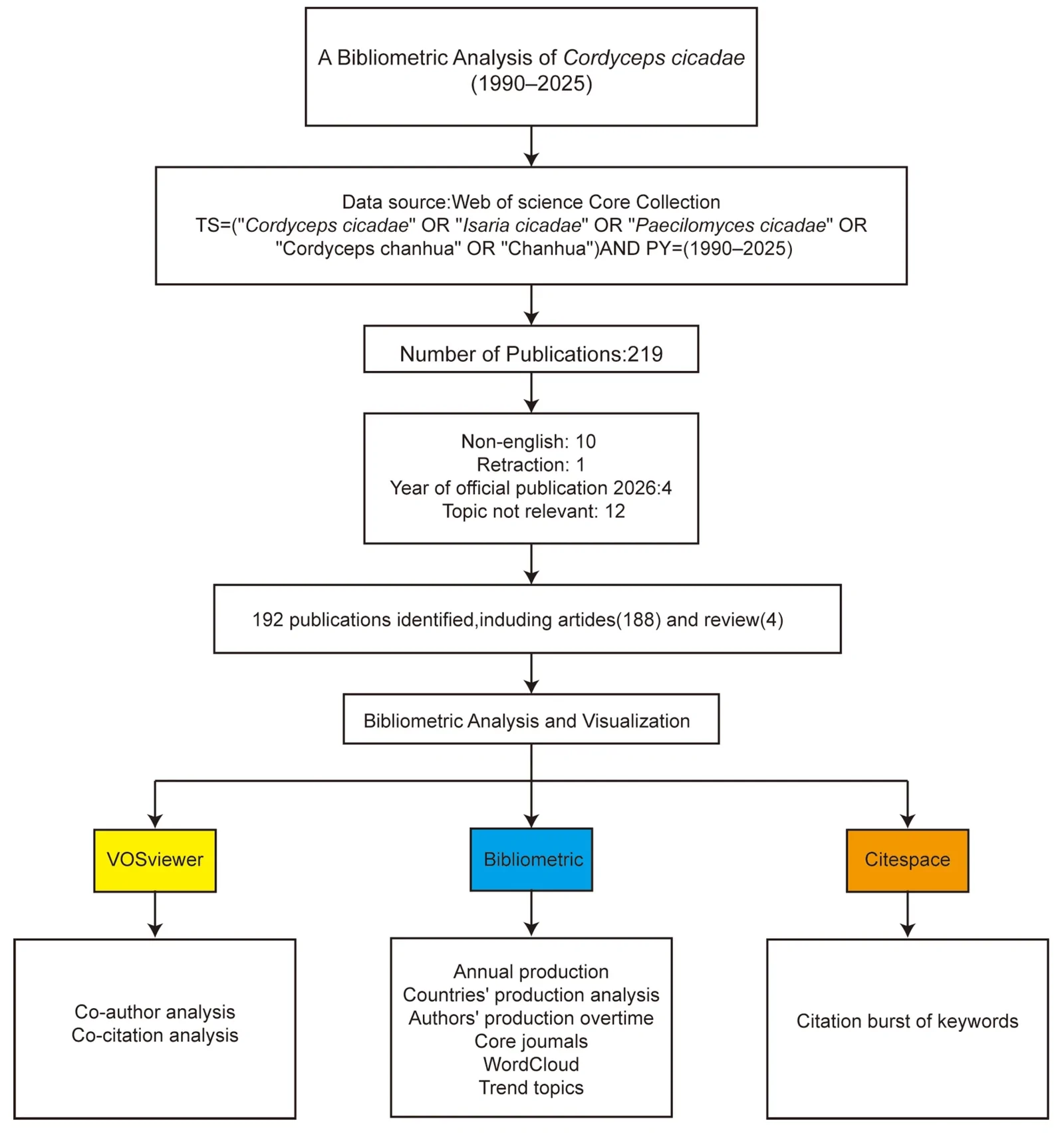
Flowchart of the literature search process in the study.

In this study, *Cordyceps cicadae* is retained as the principal literature-based term for the bibliometric analysis. The search includes historical scientific names, current scientific names, *Cordyceps chanhua*, and the vernacular term “chanhua” to capture relevant publications across changes in nomenclature and terminology. The search was conducted in September 2026 for publications dated through 2025. TS = (“*Cordyceps cicadae*” OR “*Isaria cicadae*” OR “*Paecilomyces cicadae*” OR “*Cordyceps chanhua*” OR “Chanhua”) AND PY = (1990–2025). The PY field was used to restrict the search to publication years from 1990 to 2025. The initial search retrieved 219 records. After excluding 10 non-English records, 209 English-language articles and reviews remained. During subsequent screening, one retracted publication and four records that had been published as Early Access in 2024 or 2025 but subsequently received a final publication year of 2026 were excluded, leaving 204 records for relevance screening. Two authors screened the records to exclude publications unrelated to the study topic. Any disagreements regarding exclusion were resolved by examining the full text and discussing the study’s relevance until consensus was reached. A further 12 records were excluded because they were unrelated to the study topic. The final dataset comprised 192 publications, including 188 articles and four reviews. The selection process is presented in Figure 8.

### 4.2. Bibliometric Analysis

Microsoft Excel 2021 (Microsoft Corporation, Redmond, WA, USA) was used to summarize annual publication outputs and cumulative publications.

Bibliometric analyses were performed using the Bibliometrix package (version 4.3.3) in R (version 4.5.0) to evaluate the productivity of countries, authors, and journals, as well as research trends and thematic patterns. Biblioshiny, the web-based interface of Bibliometrix, was launched using the biblioshiny() function. Bibliographic records retrieved from the Web of Science Core Collection (WoSCC), including full records and cited references, were imported for analysis. Author and journal productivity were assessed based on publication counts. Bradford’s Law analysis was conducted using the corresponding Biblioshiny module, with journals classified into three zones to identify core publication sources. Trend Topics analysis was performed for the period 1990–2025 using Keywords Plus as the analytical field, with a minimum word frequency of five and three terms displayed per year. Thematic analysis was conducted using the corresponding Biblioshiny module to identify major research themes and their relationships based on keyword co-occurrence. The analytical settings, including the keyword field, minimum frequency threshold, clustering method, and thematic mapping parameters, were specified according to the actual configurations used in Biblioshiny to ensure methodological transparency and reproducibility.

VOSviewer (version 1.6.20, Leiden University, Leiden, the Netherlands) was employed to construct and visualize collaboration networks among authors, as well as co-citation networks of journals and references. Network visualization, overlay visualization, and density visualization were generated to identify influential contributors and research hotspots.

CiteSpace (version 6.3.R1, Drexel University, Philadelphia, PA, USA) was used to perform keyword burst detection.

### 4.3. Parameter Settings

For CiteSpace, the time slice was set from 1990 to 2025 with one year per slice. The g-index (k = 25) was selected as the node selection criterion. Full counting was used to calculate publication output for countries/regions and authors, with each publication counted once for every contributing country/region and each co-author.

### 4.4. GenAI Disclosure

During the preparation of this work, the author(s) did not use any generative artificial intelligence (GenAI) tools for the generation of text, data, or graphics. All content is original and written by the authors.

## 5. Conclusions

Overall, the bibliometric findings indicate that *Cordyceps cicadae* has evolved from a traditional medicinal fungus with empirical applications into an important research subject integrating natural product chemistry, pharmacology, microbiology, biotechnology, and systems biology. Continuous advances in cultivation technologies, bioactive constituent discovery, molecular pharmacology, and microbiota-mediated mechanisms are expected to further promote its clinical translation and industrial application. This study not only provides a comprehensive overview of the global research landscape of *C. cicadae*, but also offers valuable guidance for future investigations on cultivation optimization, quality evaluation, bioactive compound discovery, mechanistic exploration, and evidence-based clinical application.

## Figures and Tables

**Table 1:** The similarities and differences between *Cordyceps cicadae* and *Cordyceps sinensis*.

Name	*Cordyceps cicadae*	*Cordyceps sinensis*
Source	It grows inside the nymph of cicada hosts and forms fruiting bodies on the surfaces of cicadas	It grows inside the larvae of lepidoptera and forms fruiting bodies on the surfaces of lepidoptera
Current name	*Isaria cicadae *Miq.,* Bull . Sci. phys. nat. néerl.*: 86 (1838 ) [MB#204858]	*Ophiocordyceps sinensis *(Berk.) G.H. Sung, J.M. Sung, Hywel-Jones & Spatafora,* Stud. Mycol. *57: 46 (2007) [MB#504340]
Homotypic synonym	*Paecilomyces cicadae* (Miq.) Samson,* Stud. Mycol. *6: 52 (1974) [MB#319102],* Cordyceps cicadae *(Miq.) Massee,* Ann. Bot. (Oxford) *9: 38 (1895) [MB#311793]	*Cordyceps sinensis *(Berk.) Sacc.,* Michelia *1 (3): 320 (1878) [MB#215164]
Classification	Fungi, Dikarya, Ascomycota, Pezizomycotina, Sordariomycetes, Hypocreomycetidae, Hypocreales, Cordycipitaceae, Isaria, Isaria cicadae	Fungi, Dikarya, Ascomycota, Pezizomycotina, Sordariomycetes, Hypocreomycetidae, Hypocreales, Ophiocordycipitaceae, Ophiocordyceps, Ophiocordyceps sinensis
Area of distribution	Produced in Zhejiang, Sichuan, Anhui, Fujian and other places.	The high-altitude habitats of the Tibetan Plateau.
Common ingredients	Nucleosides, polysaccharides, sterols, fatty acids, inorganic elements, etc.	
Characteristic component	N6-(2-hydroxyethyl) adenosine (HEA), ergosterol	
Pharmacological action	Renoprotective, hepatoprotective, anti-inflammatory, antioxidant, neuroprotective, and gut microbiota-modulating activities	Renoprotective, hepatoprotective, neuroprotective, anti-inflammatory, and antioxidant activities
References	[6,8]	[9,10]

Note: Current name and synonymy were verified using MycoBank. The taxonomic classification follows the current name listed in MycoBank rather than the historical or synonymous names used in the literature.

**Table 2:** The distribution of the authors’ productivity.

Documents Written	Number of Authors (N)	Proportion of Authors (%)
1	713	76.6%
2	135	14.5%
3	43	4.6%
4	13	1.4%
5	12	1.3%
6	6	0.6%
7	5	0.5%
8	2	0.2%
9	1	0.1%
15	1	0.1%

**Table 3:** The top 6 authors in terms of the number of published papers.

Rank	Author	Documents	Citations	Average Citations Per Publication
1	Chen, Chin-Chu	15	394	26.3
2	Hsu, Jui-Hsia	9	112	12.4
3	Li, Wei	8	230	28.8
4	Ouyang, Zhen	8	435	54.4
5	Tian, Juanjuan	7	190	27.1
6	Wang, Xiaomeng	7	190	27.1

**Table 4:** Bradford’s Law zone distribution.

Zone	Number of Journals	Number of Papers	Average Papers Per Journal
Zone 1 (Core)	10	64	6.4
Zone 2	37	65	1.8
Zone 3	63	63	1

**Table 5:** The top 5 journals in terms of the number of published papers.

Rank	Source	Publications	Citations	Average Citations Per Publication
1	International Journal of Medicinal Mushrooms	12	142	11.8
2	Frontiers in Microbiology	9	87	9.7
3	International Journal of Biological Macromolecules	9	434	48.2
4	Molecules	7	180	25.7
5	Journal of Functional Foods	6	251	41.8

**Table 6:** The Top 5 Co-Cited References.

Rank	Title	Year	Citations
1	Can *Cordyceps cicadae* be used as an alternative to *Cordyceps militaris* and *Cordyceps sinensis*?—A review	2020	45
2	N6-(2-Hydroxyethyl)adenosine in the Medicinal Mushroom *Cordyceps cicadae* Attenuates Lipopolysaccharide-Stimulated Pro-inflammatory Responses by Suppressing TLR4-Mediated NF-κB Signaling Pathways	2015	40
3	Immunomodulatory functions of extracts from the Chinese medicinal fungus *Cordyceps cicadae*	2002	35
4	Ergosterol peroxide from *Cordyceps cicadae* ameliorates TGF-β1-induced activation of kidney fibroblasts	2014	31
5	Distribution of Nucleosides in Populations of *Cordyceps cicadae*	2014	31

## Data Availability

The authors will make the raw data supporting the conclusions of this article available, without any undue reservation.
